# Numerical Study of the Environmental and Economic System through the Computational Heuristic Based on Artificial Neural Networks

**DOI:** 10.3390/s21196567

**Published:** 2021-09-30

**Authors:** Kashif Nisar, Zulqurnain Sabir, Muhammad Asif Zahoor Raja, Ag Asri Ag Ibrahim, Samy Refahy Mahmoud, Mohammed Balubaid, Danda B. Rawat, Joel J. P. C. Rodrigues

**Affiliations:** 1 Faculty of Computing and Informatics, Universiti Malaysia Sabah, Jalan UMS, Kota Kinabalu Sabah 88400, Malaysia; awgasri@ums.edu.my; 2 Department of Mathematics and Statistics, Hazara University, Mansehra 21120, Pakistan; zulquain_maths@hu.edu.pk; 3 Future Technology Research Center, National Yunlin University of Science and Technology, 123 University Road, Section 3, Douliou 64002, Taiwan; rajamaz@yuntech.edu.tw; 4 Department of Industrial Engineering, Faculty of Engineering, King Abdulaziz University, Jeddah 21589, Saudi Arabia; srhassan@kau.edu.sa; 5 GRC Department, Faculty of Applied studies, King Abdulaziz University, Jeddah 21589, Saudi Arabia; mbalubaid@kau.edu.sa; 6 Data Science and Cybersecurity Center, Deptartment of Electrical Engineering and Computer Science, Howard University, Washington, DC 20059, USA; db.rawat@ieee.org; 7 Department of Electrical Engineering, Federal University of Piauí (UFPI), Teresina 64049-550, Brazil; joeljr@ieee.org; 8 Instituto de Telecomunicações, 6201-001 Covilhã, Portugal

**Keywords:** environmental and economic system, interior-point, artificial neural networks, nonlinear model, statistical studies

## Abstract

In this study, the numerical computation heuristic of the environmental and economic system using the artificial neural networks (ANNs) structure together with the capabilities of the heuristic global search genetic algorithm (GA) and the quick local search interior-point algorithm (IPA), i.e., ANN-GA-IPA. The environmental and economic system is dependent of three categories, execution cost of control standards and new technical diagnostics elimination costs of emergencies values and the competence of the system of industrial elements. These three elements form a nonlinear differential environmental and economic system. The optimization of an error-based objective function is performed using the differential environmental and economic system and its initial conditions. The optimization of an error-based objective function is performed using the differential environmental and economic system and its initial conditions.

## 1. Introduction

Management based on the supply chain has attracted the researcher’s community in recent years due to wide ranging applications in industrial organizations from raw material to final product distribution to clients. Conceptual investigations on supply chain organization stressed the significance of the strategic associations between corporations in order to increase the operational and financial presentation of these companies to reduce the total inventories and cost in the supply chain. The principle of this association is concerned with matching between the participants [1,2,3]. Association between industry groups is an increasingly mutual avenue for these societies to maintain and find modest advantage [4,5]. The inter-firm partnering nature in the management of the supply chain was discussed by Mentzer et al. [6]. An increasing quantity of industrial groups started to realize the tactical significance of controlling, designing, and planning, supply chain systems overall rather than disconnected subsystems collection. Min et al. [7] studied the modeling supply chain processes and identified key opportunities and challenges to model the operations in supply chain networks.

To predict the growth of unified economies, the question arises whether low level economies of growth may not undergo economic losses at that time when the leading economies of the world are suffering harms in the case of economic disaster. The procedures of mathematical design are implemented for the researchers to indicate the systems as a “predator-victim” [8], which allows to form mathematical networks to define their reports. The mathematical representations of the environmental and economic system have three compartments, execution cost of control standards and new technical diagnostics (*X*), elimination costs of emergencies values (*Y*), and the competence of the system of industrial elements (*Z*). The construction of a mathematical differential system describes how to transform the corresponding variables per unit time in order to assume the nature of the quantities relationship, given as [9]:

(1) $$\left\{ \begin{array}{cc} \frac{dX}{ds}=K_{1}X(s)\left(a-X(s)\right)-K_{2}Y(s)+K_{3}Z(s), & X(0)=C_{1},\\ \frac{dY}{ds}=K_{4}X(s)\left(a-X(s)\right)-K_{5}Y(s)\left(b-Y(s)\right)+K_{6}Z(s), & Y(0)=C_{2},\\ \frac{dZ}{ds}=K_{7}X(s)-K_{8}Y(s), & Z(0)=C_{3}. \end{array}\right.$$

In the above system, *K_i_* shows the values of coefficient constant. The aim of the present study is to investigate the numerical performances of the environmental and economic system using the capabilities of the heuristic global search genetic algorithm (GA) and the quick local search interior-point algorithm (IPA) to optimize the artificial neural networks (ANNs) models, i.e., ANN-GA-IPA. The stochastic computing approaches have great competency to solve the nonlinear differential system. Few recent applications of the stochastic computing solvers are summarized in Table 1.

By impressing the cited applications of Table 1, the authors are motivated to solve the environmental and economic nonlinear system by using the computational ANN-GA-IPA. Few novel features of the computational ANN-GA-IPA in terms of objectives of the study are briefly provided as:

- To solve the nonlinear environmental and economic system successfully by novel implementation of the computational numerical heuristics of ANN-GA-IPA.
- To certify the computational procedures of ANN-GA-IPA, the consistent, stable, and robust results with reasonable accuracy should be attained for the environmental and economic nonlinear systems.
- To verify the dependability of the computational procedure of ANN-GA-IPA, the absolute error (AE) levels lie in good ranges from reference state of the art number solution of Adams method.
- To endorsement of the scheme by calculating the different statistical inferences for solving the environmental and economic nonlinear system on multiple autonomous runs of the computational procedure of ANN-GA-IPA.

The remaining parts of the paper parts are categorized as: Section 2 describes the ANN-GA-IPA procedures using the statistical presentations. Section 3 validates the result and discussions. Section 4 shows the concluding remarks and future research reports.

## 2. Methodology

The methodology section is presented in two phases; firstly, the design of ANN-GA-IPA, and secondly, the application procedure of designed ANN-GA-IPA to the environment and economic system (1). Meanwhile, the performance measures are also provided for better analysis of accuracy of the proposed computational design of ANN-GA-IPA for solving the nonlinear environmental and economic system.

### 2.1. Design of ANN-GA-IPA

The design of the proposed computing ANN-GA-IPA is presented in terms of ANN topology construction involving the input, output and hidden layers, number of hidden neurons, activation function, parameters influencing the training performance relevant to the global search of GAs and local search of IPA. A good source of reference for setting the appropriate design is provided by Ojha et al., in [25].

The mathematical performances of the nonlinear environmental and economic model are selected in three phases, execution cost of control standards and new technical diagnostics, elimination costs of emergencies values and the competence of the system of industrial elements together with their derivatives in three layers’ structure of ANNs in the form of continuous mapping are written as follows: [26,27,28]

(2) $$[\hat{X}(s),\;\;\hat{Y}(s),\;\hat{Z}(s)]=\left[\begin{array}{l} \sum_{r\;=\;1}^{k}n_{X,r}q(w_{X,r}s+l_{X,r}),\sum_{r\;=\;1}^{k}n_{Y,r}q(w_{Y,r}s+l_{Y,r}),\\ \sum_{r=\;1}^{k}l_{Z,r}q(w_{Z,r}s+l_{Z,r}) \end{array}\right],\;[{\hat{X}}^{\prime }(s),\;\;{\hat{Y}}^{\prime }(s),\;{\hat{Z}}^{\prime }(s)]=\left[\begin{array}{l} \sum_{r\;=\;1}^{k}n_{X,r}q^{\prime }(w_{X,r}s+l_{X,r}),\sum_{r\;=\;1}^{k}n_{Y,r}q^{\prime }(w_{Y,r}s+l_{Y,r}),\\ \sum_{r=\;1}^{k}n_{Z,r}q^{\prime }(w_{Z,r}s+l_{Z,r}) \end{array}\right],$$

where ***W*** is an unknown weight vector with its components depend upon number of reuron *k*. The mathematical representation of ***W*** is shown mathematically as follows:

$$W=[W_{X},\;W_{Y},W_{Z}],\;\mathrm{for}\;W_{X}=[n_{X},w_{X},l_{X}],\;W_{Y}=[n_{Y},\omega_{Y},l_{Y}],\;\mathrm{and}\;W_{Z}=[n_{Z},\omega_{Z},l_{Z}],\;\mathrm{where}\;\begin{array}{l} n_{X}=\;\;[n_{X,1},n_{X,2},\ldots ,n_{X,r}],\;\;\;\;\;\;\;\;\;\;n_{Y}=\;\;[n_{Y,1},n_{Y,2},\ldots ,n_{Y,r}],\;\;\;\;\;\;\;\;\;n_{Z}=\;\;[n_{Z,1},n_{Z,2},\ldots ,n_{Z,r}].\\ w_{X}=\;\;[w_{X,1},w_{X,2},\ldots ,w_{X,r}],\;\;w_{Y}=\;\;[w_{Y,1},w_{Y,2},\ldots ,w_{Y,r}],\;\;\;w_{Z}=\;\;[w_{Z}{}_{,1},w_{Z}{}_{,2},\;\ldots ,w_{Z}{}_{,r}],\;\;\;\;\;\;\;\\ l_{X}=\;\;[l_{X,1},l_{X,2},\ldots ,l_{X,r}],\;\;\;\;\;\;\;\;\;\;\;\;\;\;\;l_{Y}=\;\;[l_{Y,1},l_{Y,2},\ldots ,l_{Y,r}],\;\;\;\;\;\;\;\;\;\;\;\;\;\;\;\;\;\;l_{Z}=\;\;[l_{Z,1},l_{Z,2},\ldots ,l_{Z,r}]. \end{array}$$

A large verity of activation functions, we have chosen Log-Sigmoid $q(s)={\left(1+e^{-s}\right)}^{-1}$ in the presented study and applied to Equation (2), then we have:

(3) $$\begin{array}{l} {}[\hat{X}(s),\;\hat{Y}(s),\;\hat{Z}(s)]=\left[\begin{array}{l} \sum_{r=1}^{m}\frac{n_{X,r}}{1+e^{-\left(w_{X,r}s+l_{X,r}\right)}},\;\;\;\frac{n_{Y,r}}{1+e^{-\left(w_{Y,r}s+l_{Y,r}\right)}},\\ \sum_{r=1}^{m}\frac{n_{Z,r}}{1+e^{-\left(w_{Z,r}s+l_{Z,r}\right)}},\;\; \end{array}\right],\\ {}[{\hat{X}}^{\prime }(s),\;{\hat{Y}}^{\prime }(s),\;{\hat{Z}}^{\prime }(s)]=\left[\begin{array}{l} \sum_{r=1}^{m}\frac{n_{X,r}w_{X,r}e^{-\left(w_{X,r}s+i_{X,r}\right)}}{{\left(1+e^{-\left(w_{X,r}s+l_{X,r}\right)}\right)}^{2}},\;\sum_{r=1}^{m}\frac{n_{Y,r}w_{Y,r}e^{-\left(w_{Y,r}s+i_{Y,r}\right)}}{{\left(1+e^{-\left(w_{Y,r}s+l_{Y,r}\right)}\right)}^{2}},\\ \sum_{r=1}^{m}\frac{n_{Z,r}w_{Z,r}e^{-\left(w_{Z,r}s+i_{Z,r}\right)}}{{\left(1+e^{-\left(w_{Z,r}s+l_{Z,r}\right)}\right)}^{2}} \end{array}\right]. \end{array}$$

The appropriate arrangement of networks in Equation (3) can be exploited to solve differential systems as represented in (1) with the availability of suitable *W*. The ANNs parameters are optimized with combined strength of global and local search methodologies of GAs and IPA, i.e., ANN-GA-IPA.

GA is a famous global search procedure of optimization work to solve both linear and nonlinear systems. GA is typically used to control the results of the precise population for solving various steep/complicated models based on optimal training. In recent years, GA is implemented in the shotgun metabolomics [29], wellhead back pressure control system [30], bearing fault diagnosis of induction motors [31], energy efficient clustered wireless sensor networks [32], beam deflection monitoring systems [33], adjustment problem of sensor acquisition frequency [34], image processing optimization tasks [35], and torque adjustment for the ankle push-off in the walking bipedal robots [36]. The optimization performance in terms of efficiency, accuracy, and viability of GAs is further enhanced by introducing the concept of hybridization with efficient local search.

*Interior point algorithm* is a one of the local search optimization approaches generally applied to solve both types of constrained/unconstrained models in optimization tasks. It is a well-organized algorithm used to compute the results competently. Recently, IPA is a reliable treatment of the economic load dispatch problem [37], dynamic adjustments of step sizes and tolerances [38], active noise control systems [39], convex quadratic programming [40], and optimization of models representing the dynamics of heartbeat [41].

### 2.2. Application ANN-GA-IPA to Environment and Economic System

In this section, the application procedure of ANN-GA-IPA is presented for the environmental and economic nonlinear model in terms of fitness function developments, formulation of pseudo code, and workflow of the procedural steps.

An objective function is presented as:

(4) $$E=\sum_{r=i}^{4}E_{r}$$

(5) $$E_{1}=\frac{1}{N}\sum_{r=1}^{N}{\left[{\hat{T}}_{r}^{\prime }+a{\hat{F}}_{r}{\hat{T}}_{r}+b\right]}^{2},\;$$

(6) $$E_{2}=\frac{1}{N}\sum_{r=1}^{N}{\left[{\hat{F}}_{r}^{\prime }-d{\hat{T}}_{r}-c{\hat{M}}_{r}{\hat{F}}_{r}\right]}^{2},\;$$

(7) $$E_{3}=\frac{1}{N}\sum_{r=1}^{N}{\left[{\hat{M}}_{r}^{\prime }+d{\hat{T}}_{r}-b{\hat{F}}_{r}{\hat{M}}_{r}+\delta \right]}^{2},\;$$

(8) $$E_{4}=\frac{1}{3}\left[{\left({\hat{T}}_{0}-r_{1}\right)}^{2}+{\left({\hat{F}}_{0}-r_{2}\right)}^{2}+{\left({\hat{M}}_{0}-r_{3}\right)}^{2}\right],$$

where ${\hat{X}}_{r}=X\left(s_{r}\right),\;\;{\hat{Y}}_{r}=Y\left(s_{r}\right),\;{\hat{Z}}_{r}=Z\left(s_{r}\right),\;\;\;Nh=1,\;$ and $\;s_{r}=hr$, ${\hat{X}}_{r}$, ${\hat{Y}}_{r}$ and ${\hat{Z}}_{r}$ indicate the proposed outcomes of ANN-GA-IPA for execution cost of control standards and new technical diagnostics, elimination costs of emergencies values, and the competence of the system of industrial elements, respectively, i.e., indicator of environmental and economic systems. Accordingly, Equations (5)–(7) signify objective functions using indicators of nonlinear environmental and economic models while Equation (8) represents an objective function based on the initial conditions.

**Figure 1:**
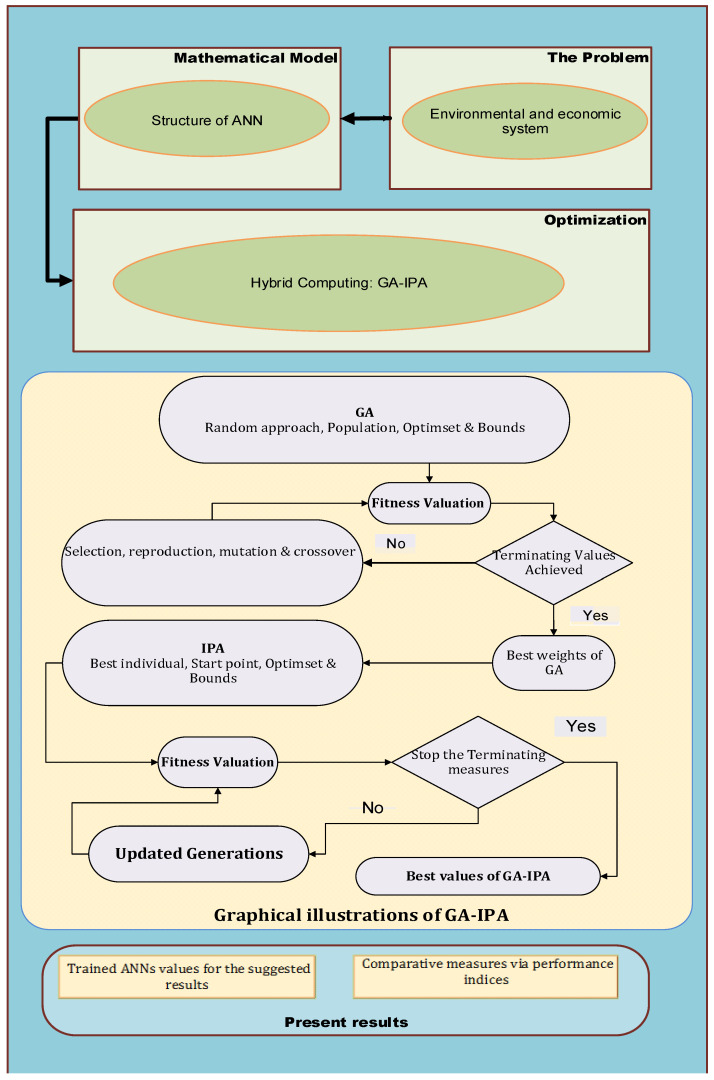
Design procedure of ANN-GA-IPA to solve the nonlinear environmental and economic model.

The optimization of the ANN model-based fitness function (5)–(8) conducted initially with GAs for the global search and performance of GAs by mean of efficiency in computational time is further enhanced by the procedure of IPA-based rapid local search. In other words, heuristic GA-IPA is implemented for the global search (exploration, GA) and effectiveness to exploit a solution (IPA, intensification), for finding the decision variables of ANN models of the environmental and economic system. The pseudo code for implementation of GA-IPA is narrated in Algorithm 1, while the procedural steps involved in implementation of ANN-GA-IPA are shown in Figure 1 for solving the environmental and economic systems.
**Algorithm 1.** Pseudo code for optimization for the environmental and economic nonlinear model by ANN-GA-IPA. **Start GA** **Inputs:** To measure the chromosomes of the same network element as: ***W*** = [n,w,l] **Population:** Set of chromosome is given as: $W_{X}=[n_{X},\omega_{X},l_{X}]$, $W_{Y}=[n_{Y},\omega_{Y},l_{Y}]$ and $W_{Z}=[n_{Z},\omega_{Z},l_{Z}]$. **Output:** Global weight values are ***W*_GB_** **Initialization:** To adjust the chromosomes selection, adjust the ***W*_GB_**. **Fit Estimation:** Modify the values of FIT (*E*) using the  population (***P***) for systems 4 to 8 **Stopping standards:** Terminate if [Iterations = 60], [*E* = 10^−06^], [TolCon = 10^−10^], [StallLimit = 120], [TolFun = 10^−10^] & [PopSize = 200].Go to **storage** **Ranking:** For the FIT (*E*), rank ***W*_GB_** in population. **Storage:** Store ***W*_GB_**, time, iterations, *E* & count of function for the  GA. **GA process Ends** **IPA Starts** **Inputs:**
***W*_GB_** is the Start point: **Output:** The best GAIPA weights are represented as ***W*_GIPA_**  **Initialize:** Iterations, Assignments & ***W*_GB_**. **Terminating Standards:** Stop, if [*E* = 10^−12^], [MaxFunEvals = 100,000], [TolX = 10^−12^], [TolFun = 10^−12^] & [Iterations = 500]. **FIT approximation:** Compute FIT & ***W*_GIPA_** using Equations (4) to (8). **Amendments:** Regulate ‘fmincon’ for the values of IPA, *E* to  improve the ‘***W***’ for Equations (4)–(8). **Accumulate:** Transform ***W*_GIPA_**, function counts, time FIT,  iterations for the IPA present runs. **IPA End**

### 2.3. Performance Measures

The statistical measures using the mean absolute deviation (MAD), semi interquartile range (S.I.R), variance account for (VAF), and Theil’s inequality coefficient (TIC) using their global demonstrations to solve the environmental and economic nonlinear model is mathematically written as:

(9) $${[\mathrm{MAD}}_{X},\;{\mathrm{MAD}}_{Y}{,\;\mathrm{MAD}}_{Z}]=\left[\sum_{r=1}^{n}\left|X_{r}-{\hat{X}}_{r}\right|,\sum_{r=1}^{n}\left|Y_{r}-{\hat{Y}}_{r}\right|,\sum_{r=1}^{n}\left|Z_{r}-{\hat{Z}}_{r}\right|\right],$$

(10) $$\left\{ \begin{array}{l} S.I.R=-\frac{1}{2}\times \left(q_{1}-q_{3}\right),\\ q_{1}=1st\;\mathrm{quartile}\;\;\&\;\;\;q_{3}\;=3rd\;\mathrm{quartile},\; \end{array}\right.$$

(11) $$\left[{\mathrm{TIC}}_{X},{\mathrm{TIC}}_{Y},{\mathrm{TIC}}_{Z}\right]=\left[\begin{array}{l} \frac{\sqrt{\frac{1}{n}{\sum_{r=1}^{n}\left(X_{r}-{\hat{X}}_{r}\right)}^{2}}}{\left(\sqrt{\frac{1}{n}\sum_{r=1}^{n}{\hat{X}}_{r}^{2}}+\sqrt{\frac{1}{n}\sum_{r=1}^{n}{\hat{X}}_{r}^{2}}\right)},\\ \frac{\sqrt{\frac{1}{n}{\sum_{r=1}^{n}\left(Y_{r}-{\hat{Y}}_{r}\right)}^{2}}}{\left(\sqrt{\frac{1}{n}\sum_{r=1}^{n}{\hat{Y}}_{r}^{2}}+\sqrt{\frac{1}{n}\sum_{r=1}^{n}{\hat{Y}}_{r}^{2}}\right)},\\ \frac{\sqrt{\frac{1}{n}{\sum_{r=1}^{n}\left(Z_{r}-{\hat{Z}}_{r}\right)}^{2}}}{\left(\sqrt{\frac{1}{n}\sum_{r=1}^{n}{\hat{Z}}_{r}^{2}}+\sqrt{\frac{1}{n}\sum_{r=1}^{n}{\hat{Z}}_{r}^{2}}\right)}, \end{array}\right],$$

(12) $$\left\{ \begin{array}{l} {[\mathrm{VAF}}_{X},{\mathrm{VAF}}_{Y},{\mathrm{VAF}}_{Z}]=\left[\begin{array}{l} \left(1-\frac{\mathrm{var}\left(X_{r}-{\hat{X}}_{r}\right)}{\mathrm{var}\left(X_{r}\right)}\right)*100,\\ \left(1-\frac{\mathrm{var}\left(Y_{r}-{\hat{Y}}_{r}\right)}{\mathrm{var}\left(Y_{r}\right)}\right)*100,\\ \left(1-\frac{\mathrm{var}\left(Z_{r}-{\hat{Z}}_{r}\right)}{\mathrm{var}\left(Z_{r}\right)}\right)*100, \end{array}\right]\\ {[\mathrm{EVAF}}_{X},{\mathrm{EVAF}}_{Y},{\mathrm{EVAF}}_{Z}]=\left[\left|100-{\mathrm{VAF}}_{X},100-{\mathrm{VAF}}_{Y},100-{\mathrm{VAF}}_{Z}\right|\right]. \end{array}\right.,$$

where $\hat{X}$, $\hat{Y}$, and $\hat{Z}$ signify the approximate results.

## 3. Results of Simulations

In this section, the environmental and economic nonlinear model presented in the system (1) is numerically performed by using the computational ANN-GA-IPA. The obtained numerical outcomes of the environmental and economic nonlinear model are compared with the Adams result. The plots of AE, convergence analysis, and performance measures through different operatives are also presented. The simplified form of the environmental and economic nonlinear model using appropriate parameters is given as:

(13) $$\left\{ \begin{array}{l} \frac{dX}{ds}=0.2X(s)\left(10-X(s)\right)-0.3Y(s)+0.4Z(s),\;\;\;X(0)=2,\\ \frac{dY}{ds}=0.2X(s)\left(10-X(s)\right)+0.3Y(s)\left(5-Y(s)\right)+0.3Z(s),\;\;Y(0)=4,\\ \frac{dZ}{ds}=0.4X(s)-0.3Y(s),\;\;\;Z(0)=3. \end{array}\right.$$

An objective function for the environmental and economic nonlinear model given in Equation (13) is written as:

(14) $$\begin{array}{l} E=\frac{1}{N}\sum_{r=1}^{N}\left(\begin{array}{l} {\left[{\hat{X}}_{r}^{\prime }-0.2{\hat{X}}_{r}\left(10-{\hat{X}}_{r}\right)+0.3{\hat{Y}}_{r}-0.4{\hat{Z}}_{r}\right]}^{2}+\\ {\left[{\hat{Y}}_{r}^{\prime }-0.2{\hat{X}}_{r}\left(10-{\hat{X}}_{r}\right)-0.3{\hat{Y}}_{r}\left(5-{\hat{Y}}_{r}\right)-0.3{\hat{Z}}_{r}\right]}^{2}\\ +{\left[{\hat{Z}}_{r}^{\prime }-0.4X_{r}+0.3{\hat{Y}}_{r}\right]}^{2} \end{array}\right)\\ \;\;\;\;\;\;+\frac{1}{3}\left[{\left({\hat{X}}_{0}-2\right)}^{2}+{\left({\hat{Y}}_{0}-4\right)}^{2}+{\left({\hat{Z}}_{0}-3\right)}^{2}\right]. \end{array}$$

**Figure 2:**
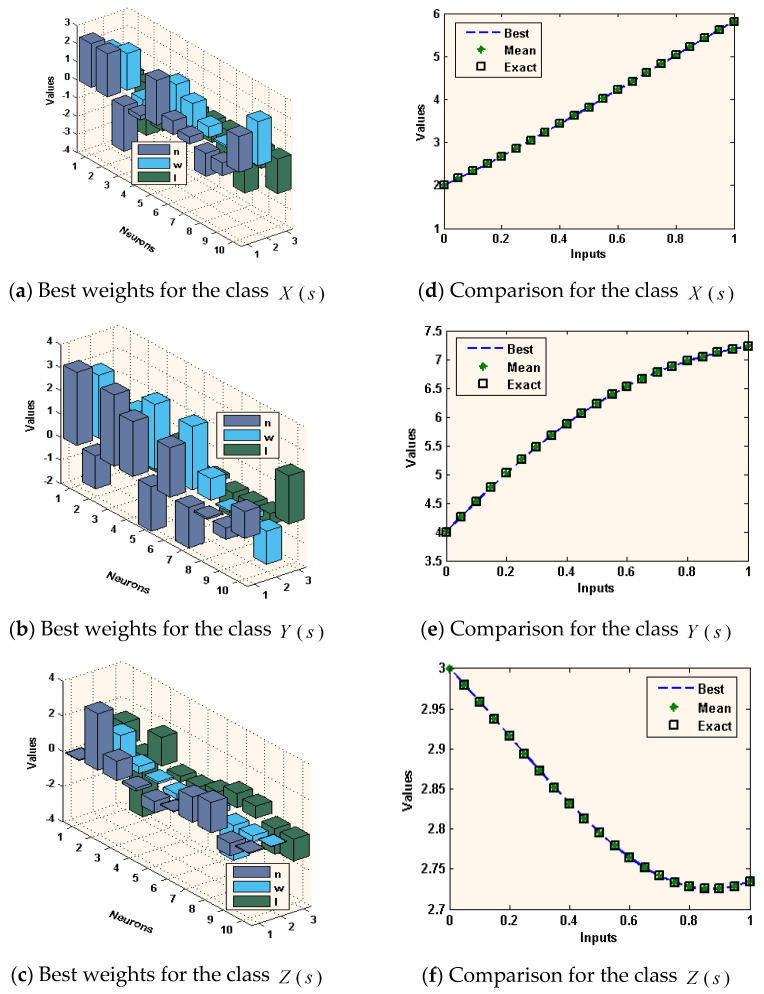
Best values of the weights vectors and results comparison to solve the environmental and economic nonlinear model.

**Figure 3:**
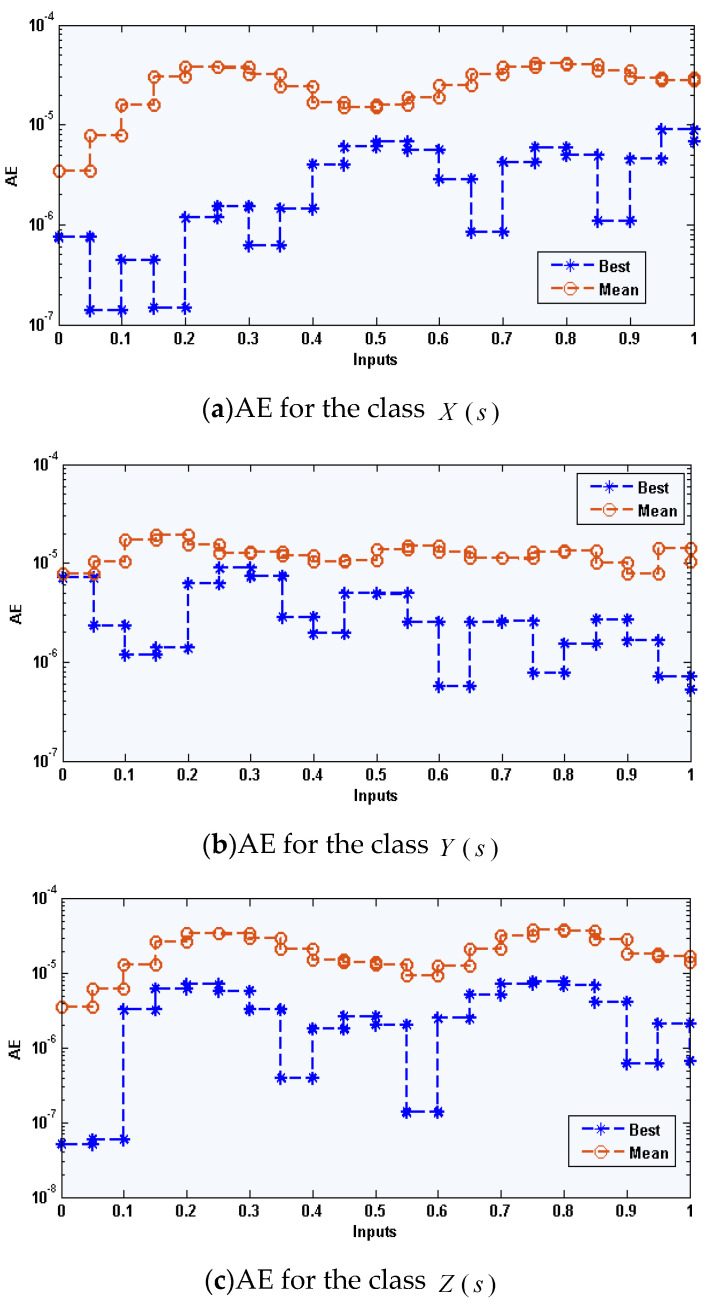
AE values for each class of the environmental and economic nonlinear model.

**Figure 4:**
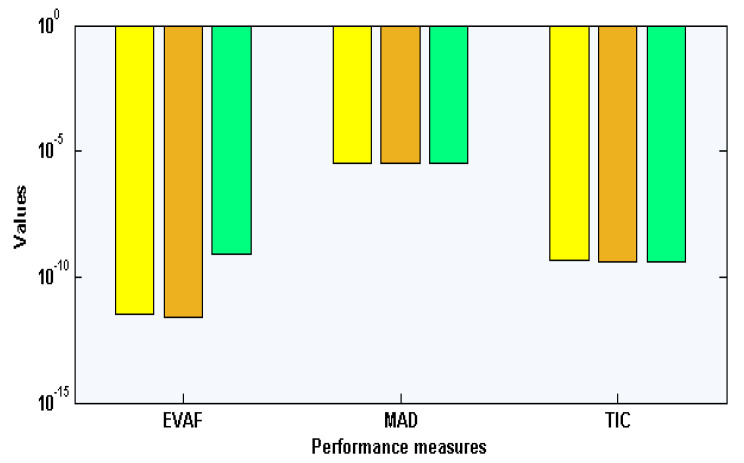
Performance indices through MAD, TIC, and EVAF for solving the environmental and economic nonlinear model.

The environmental and economic nonlinear model given in Equation (1) is applied to optimize the computational GAIPA for the ANN parameters using 30 number of variables with the step size of 0.05. The best weight vectors are derived for solving the environmental and economic nonlinear model in the below equations are given as:

(15) $$\begin{array}{l} \hat{X}(s)=\frac{2.4095}{1+e^{-(1.6650s-0.4688)}}-\frac{2.3755}{1+e^{-(2.0171s-2.8447)}}-\\ \;\;\;\;\;\;\;\;\;\;\;\frac{-2.4751}{1+e^{-(-0.8022s-2.0837)}}\;-\frac{-0.2371}{1+e^{-(\;0.428s-0.7601)}}-\\ \;\;\;\;\;\;\;\;\;\;\;\frac{2.4625}{1+e^{-(\;1.8727s-0.2840)}}-\frac{0.7465}{1+e^{-(1.3418s-1.1636)}}+\\ \;\;\;\;\;\;\;\;\;\;\;\frac{0.3902}{1+e^{-(\;0.5808s-1.8805)}}-\frac{-1.2598}{1+e^{-(\;0.0985s+2.9004)}}+\\ \;\;\;\;\;\;\;\;\;\;\;\frac{-0.7051}{1+e^{-(\;0.3106s-1.0296)}}+\frac{1.9644}{1+e^{-(2.4220s-1.8985)}}, \end{array}$$

(16) $$\begin{array}{l} \hat{Y}(s)=\frac{3.1478}{1+e^{-(2.7157s-0.0170)}}-\frac{1.4241}{1+e^{-(0.4208s+0.4282)}}-\\ \;\;\;\;\;\;\;\;\;\;\frac{3.0698}{1+e^{-(\;1.9966s-0.9828)}}-\frac{2.3612}{1+e^{-(\;2.7915s+0.7510)}}-\\ \;\;\;\;\;\;\;\;\;\;\frac{-1.9140}{1+e^{-(\;0.6910s-0.5481)}}-\frac{2.1246}{1+e^{-(2.7231s-0.1898)}}-\\ \;\;\;\;\;\;\;\;\;\;\frac{1.7418}{1+e^{-(\;0.9288s+1.0432)}}-\frac{0.0728}{1+e^{-(-0.0701s-0.7089)}}-\\ \;\;\;\;\;\;\;\;\;\;\frac{0.4869}{1+e^{-(-0.2474s-0.4771)}}+\frac{1.1565}{1+e^{-(-1.4257s+2.0715)}}, \end{array}$$

(17) $$\begin{array}{l} \hat{Z}(\Omega )=\frac{0.8294}{1+e^{-(-0.3152s+0.6433)}}-\frac{0.0758}{1+e^{-(0.0585s-0.6263)}}-\\ \;\;\;\;\;\;\;\;\;\;\;\frac{1.0697}{1+e^{-(\;0.1049s-1.7787)}}-\frac{0.8529}{1+e^{-(\;0.2868s+0.4633)}}-\\ \;\;\;\;\;\;\;\;\;\;\;\frac{0.2767}{1+e^{-(\;0.9567s+0.9975)}}-\frac{0.2788}{1+e^{-(0.5610s-0.5855)}}-\\ \;\;\;\;\;\;\;\;\;\;\;\frac{0.3144}{1+e^{-(\;0.7972s+0.1543)}}-\frac{1.2123}{1+e^{-(-0.383s+0.6628)}}-\\ \;\;\;\;\;\;\;\;\;\;\;\frac{0.5234}{1+e^{-(-0.020s+1.1699)}}+\frac{0.3777}{1+e^{-(0.4164s+2.9967)}}, \end{array}$$

where, $\hat{X}$, $\hat{Y}$, and $\hat{Z}$ are the approximate results of Equation (13) for the environmental and economic system (1) by ANN-GA-IPA using the best weights of ANNs in the first equation of set (2). Figure 2, Figure 3 and Figure 4 demonstrate the best weights performance, comparison of the results, and AE values to solve the nonlinear environmental and economic system using the computational performance of ANN-GA-IPA.

We conducted the implementation of the proposed integrated metaheuristic of ANN-GA-IPA by variants of parameters, i.e., *K_i_* for *i* = 1, 2, …, 8, and initial conditions, i.e., *C_i_* for *i* = 1, 2, 3 with almost similar objective function as shown in Equation (14) and accuracy of the results are found in the similar range/levels of precision as that of the problem of environmental and economic systems presented in (13). Therefore, to avoid the redundant representation of illustrations, we confined in this study to present the results of ANN-GA-IPA for problem (13) for single and multiple autonomous execution of ANN-GA-IPA for effective remarks on accuracy, convergence, stability, and robustness.

The best weight values for the environmental and economic nonlinear model have been established in Figure 1a–c for 10 neurons and 30 variables. These weight vectors plots are demonstrated in the above Equations (15)–(17). The comparison of the result’s performance for the control standards and new technical diagnostics, elimination costs of emergencies values, and the competence of the system of industrial elements based on the environmental and economic nonlinear model is provided in Figure 1d–f. The best and mean outcomes are derived using the ANN-GA-IPA based on the nonlinear environmental and economic system. The precise performance of the computational ANN-GA-IPA is observed for each class of the nonlinear environmental and economic model. The AE plots are derived in Figure 4a–c for each class of the environmental and economic nonlinear model. It is stated that the AE based on mean and best results is found in good trials. One can find that the best AE of the X(s), Y(s), and Z(s) based on the nonlinear environmental and economic model lie around 10-06-10-08, 10-04-10-06, and 10-05-10-08. The AE mean values for X(s), Y(s), and Z(s) lie 10-04-10-05, 10-04-10-06, and 10-05-10-06. This very good range of AE enhances the worth of the computational ANN-GA-IPA. The performance through EVAF, TIC, and MAD operators is observed in Figure 4. One can observe that the EVAF values for the control standards and new technical diagnostics, elimination costs of emergencies values, and the competence of the system of industrial elements based on the environmental and economic nonlinear model lie 10-04-10-05, 10-10-10-13, and 10-07-10-08. The MAD values for these classes lie around 10-04-10-05. The TIC values for these classes lie around 10-08-10-09, 10-09-10-10, and 10-07-10-09.

**Figure 5:**
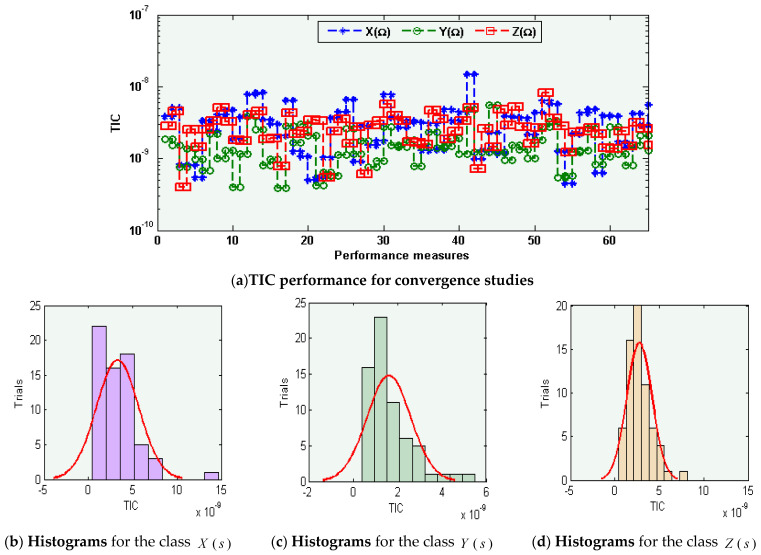

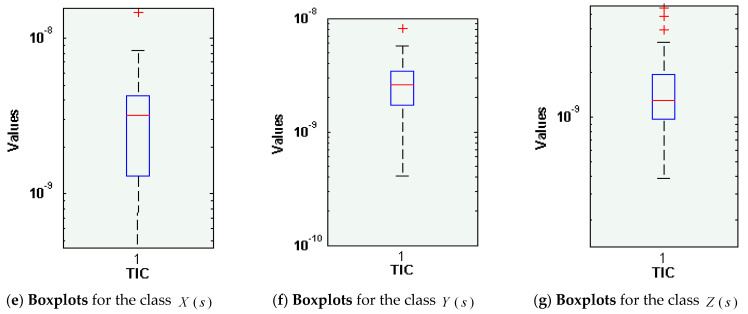
TIC operator performances based on ANN-GA-IPA to solve the environmental and economic nonlinear model.

**Figure 6:**
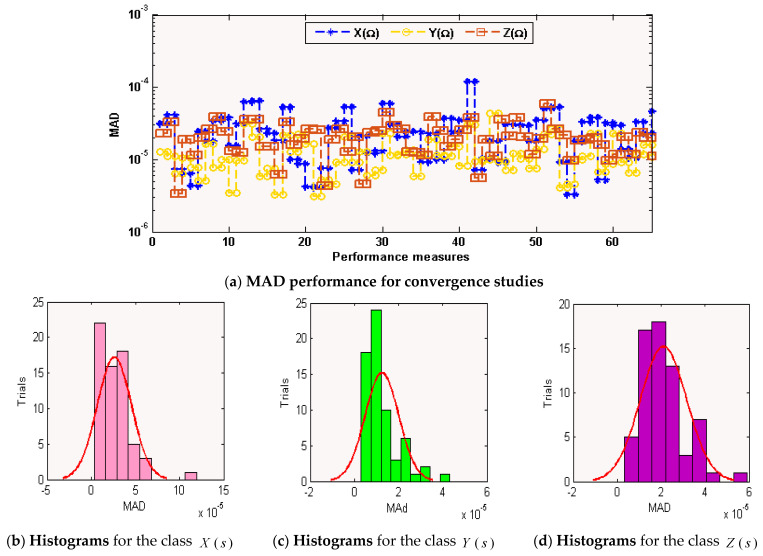

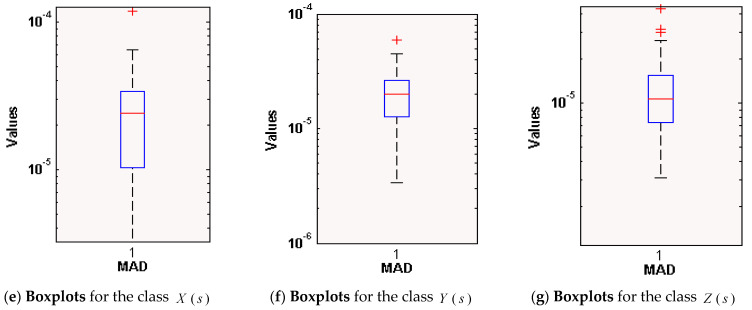
MAD operator performances based on ANN-GA-IPA to solve the environmental and economic nonlinear model.

**Figure 7:**
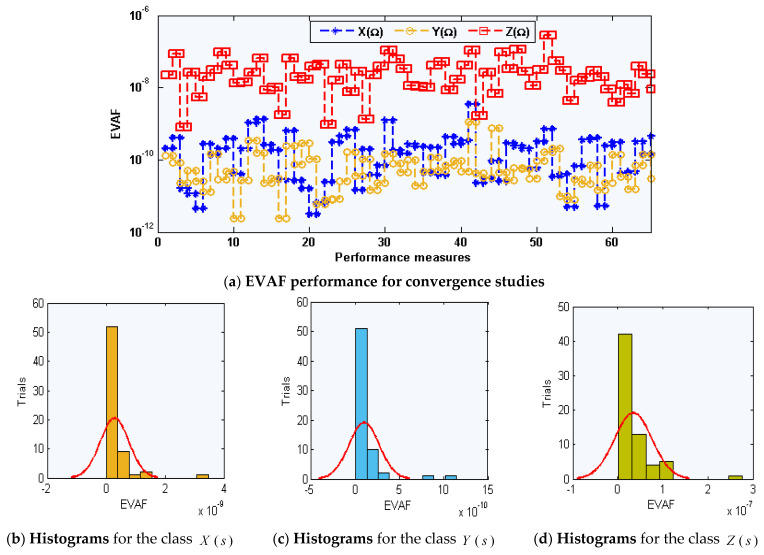

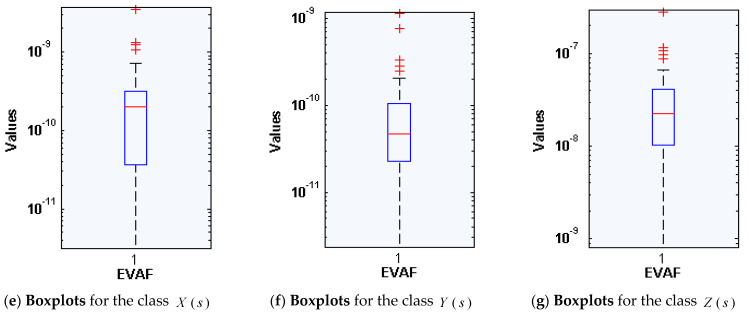
EVAF operator performances based on ANN-GA-IPA to solve the environmental and economic nonlinear model.

The graphical representations of the statistical operators along with the performances of histograms/boxplot are illustrated in Figure 5, Figure 6 and Figure 7 for solving each class of the environmental and economic nonlinear model. The convergence plots through the TIC, MAD, and EVAF operators for solving each category of the environmental and economic nonlinear model. One can see that the TIC performances lie around 10-08-10-09, 10-08-10-10, and 10-09-10-10. The MAD performances lie around 10-04-10-05, 10-04-10-06, and 10-05-10-06. Likewise, the EVAF performances lie around 10-09-10-11, 10-10-10-12, and 10-07-10-09. The achieved best performances via ANN-GA-IPA are calculated appropriately for the operators TIC, MAD, and EVAF.

For accuracy performance, statistical studies are provided in Table 2, Table 3 and Table 4 to solve the environmental and economic nonlinear model using the statistical operators based on standard deviation (SD), median (MED), S.I.R, minimum (Min), and maximum (Max). The Min values show the best performances lie around 10-07-10-09, while the Max values specify the worst result lie 10-04-10-05, the MED, Mean, S.I.R, and STD performances lie 10-05-10-06 for each class of the environmental and economic nonlinear model. One can realize performance worth through ANN-GA-IPA based on these statistical operator values lie around, in good measures, to solve the environmental and economic nonlinear model.

The global best performances of MAD, EVAF, and TIC operators for 65 trials based on the proposed computational ANN-GA-IPA are given in Table 5 to solve the environmental and economic nonlinear model. The global MED performances based on TIC, MAD, and EVAF gages lie 10-05-10-06, 10-08-10-09, and 10-10-10-11, while the S.I.R global performances of TIC, MAD, and EVAF lie 10-05-10-06, 10-09-10-10, and 10-08-10-11 to solve the environmental and economic nonlinear model. These optimal close results established the global presentations indicate the accurateness, correctness, and exactness of the proposed computational ANN-GA-IPA.

## 4. Conclusions

The purpose of this study is to treat the environmental and economic nonlinear model numerically using the ANNs strength together with the capability of global as well as local search schemes, i.e., ANN-GA-IPA. An objective function is designed on the basis of the environmental and economic nonlinear model and its boundary conditions. The optimization of the objective function based on the environmental and economic nonlinear model is performed using the ANN-GA-IPA strength. The proposed results are compared with the Adams solutions to check the correctness of the ANN-GA-IPA for solving the environmental and economic nonlinear model. The values of the AE are calculated in good measures to solve each category of the environmental and economic nonlinear model. Furthermore, the statistical operators based on MAD, TIC, and EVAF performances have been calculated accurately to solve each category of the environmental and economic nonlinear model. The assessments through statistics performances for 65 independent executions using ANN-GA-IPA for the MED, Min, S.I.R, Max, STD, and mean operators authenticate correctness and worth of the designed computational ANN-GA-IPA. The global performances via statistical processes in terms of S.I.R and MED have been efficiently applied to each category of the environmental and economic nonlinear model.

In the future, the designed computational ANN-GA-IPA is capable to solve the biological nonlinear systems, fluid dynamic systems, and singular higher order systems.

## Figures and Tables

**Table 1:** A brief literature review of the stochastic numerical solver application in a variety of fields.

Method/Algorithm	Application	References
Fractional Mayer wavelet ANNs	Nonlinear singular fractional models in astrophysics	[10,11]
Neuro-Swarm and Neuro-Evolution integrated heuristics	Nonlinear SITR system of COVID-19 Spread	[12,13]
Computational intelligence of FFANN-GASQP	Nonlinear mosquito dispersal nonlinear system	[14]
Integrating solvers via ANN-GA-SQP, ANN-GA-ASA, and ANN-PSO-IPS	Nonlinear singular Lane–Emden or Emden Fowler systems	[15,16,17]
Hybrid intelligent mechanism with ANN, Gas, and IPA.	Differential model of prey-predator system	[18]
Computational Heuristics of ANN-GA-SQP	Dengue fever model representation with nonlinear system	[19]
Intelligent computing involving FMNEICS	Nonlinear doubly singular differential systems	[20,21]
Self-adaptive global mine blast algorithm	Six different dataset representing clustering application	[22]
Supervised and unsupervised Neural networks	Different form of ordinary/partial differential equations	[23,24]

**Table 2:** Statistical presentations of the environmental and economic nonlinear model for X(s).

s	X(s)					
	MIN	MAX	MED	MEAN	S.I.R	STD
0	7.0743 × 10^−8^	4.7294 × 10^−5^	1.1257 × 10^−6^	3.4587 × 10^−6^	1.4585 × 10^−6^	7.1764 × 10^−6^
0.05	4.3791 × 10^−9^	6.2691 × 10^−5^	4.2221 × 10^−6^	7.8281 × 10^−6^	2.7543 × 10^−6^	1.1642 × 10^−5^
0.1	4.3980 × 10^−7^	8.6225 × 10^−5^	1.3970 × 10^−5^	1.5741 × 10^−5^	5.5506 × 10^−6^	1.3619 × 10^−5^
0.15	1.4628 × 10^−7^	8.6543 × 10^−5^	3.1742 × 10^−5^	2.9933 × 10^−5^	1.4389 × 10^−5^	1.9541 × 10^−5^
0.2	4.2069 × 10^−7^	1.2099 × 10^−4^	3.8358 × 10^−5^	3.7281 × 10^−5^	2.2491 × 10^−5^	2.9381 × 10^−5^
0.25	1.0399 × 10^−7^	1.6466 × 10^−4^	3.4508 × 10^−5^	3.7597 × 10^−5^	2.5150 × 10^−5^	3.3362 × 10^−5^
0.3	5.8136 × 10^−7^	1.8272 × 10^−4^	2.6808 × 10^−5^	3.2238 × 10^−5^	2.0967 × 10^−5^	3.1584 × 10^−5^
0.35	2.0488 × 10^−7^	1.7620 × 10^−4^	1.7084 × 10^−5^	2.4104 × 10^−5^	1.4178 × 10^−5^	2.7355 × 10^−5^
0.4	4.0904 × 10^−7^	1.5131 × 10^−4^	9.1530 × 10^−6^	1.6722 × 10^−5^	8.2770 × 10^−6^	2.4136 × 10^−5^
0.45	2.4855 × 10^−7^	1.1703 × 10^−4^	8.3726 × 10^−6^	1.4900 × 10^−5^	4.5145 × 10^−6^	2.1680 × 10^−5^
0.5	6.3360 × 10^−7^	1.2621 × 10^−4^	8.7141 × 10^−6^	1.5906 × 10^−5^	5.5567 × 10^−6^	2.0310 × 10^−5^
0.55	8.4288 × 10^−8^	1.2090 × 10^−4^	1.3416 × 10^−5^	1.8517 × 10^−5^	6.4564 × 10^−6^	1.9342 × 10^−5^
0.6	8.3379 × 10^−7^	9.8065 × 10^−5^	2.1939 × 10^−5^	2.4509 × 10^−5^	6.9207 × 10^−6^	1.8620 × 10^−5^
0.65	2.2875 × 10^−7^	9.6707 × 10^−5^	3.2586 × 10^−5^	3.1577 × 10^−5^	1.5737 × 10^−5^	2.1481 × 10^−5^
0.7	1.8333 × 10^−6^	1.1287 × 10^−4^	3.9546 × 10^−5^	3.7769 × 10^−5^	1.9937 × 10^−5^	2.6668 × 10^−5^
0.75	4.3106 × 10^−7^	1.2949 × 10^−4^	4.2578 × 10^−5^	4.0906 × 10^−5^	2.3594 × 10^−5^	3.1227 × 10^−5^
0.8	7.7704 × 10^−8^	1.5822 × 10^−4^	3.9862 × 10^−5^	3.9923 × 10^−5^	2.2828 × 10^−5^	3.2959 × 10^−5^
0.85	1.7169 × 10^−7^	1.9908 × 10^−4^	3.3272 × 10^−5^	3.5134 × 10^−5^	1.9972 × 10^−5^	3.2436 × 10^−5^
0.9	8.7346 × 10^−7^	2.2773 × 10^−4^	2.2861 × 10^−5^	2.9667 × 10^−5^	1.3509 × 10^−5^	3.2102 × 10^−5^
0.95	9.5136 × 10^−7^	2.3451 × 10^−4^	2.0569 × 10^−5^	2.7424 × 10^−5^	1.1005 × 10^−5^	3.3018 × 10^−5^
1	9.5870 × 10^−7^	2.1077 × 10^−4^	2.3423 × 10^−5^	2.8972 × 10^−5^	1.1057 × 10^−5^	3.0340 × 10^−5^

**Table 3:** Statistical presentations of the environmental and economic nonlinear model for Y(s).

s	Y(s)					
	MIN	MAX	MED	MEAN	S.I.R	STD
0	9.1378 × 10^−9^	1.0984 × 10^−4^	2.792 × 10^−6^	3.4587 × 10^−6^	3.6245 × 10^−6^	1.5545 × 10^−5^
0.05	2.4578 × 10^−7^	9.9356 × 10^−5^	5.3448 × 10^−6^	7.8281 × 10^−6^	5.2596 × 10^−6^	1.4870 × 10^−5^
0.1	9.3748 × 10^−7^	8.3141 × 10^−5^	1.5007 × 10^−5^	1.5741 × 10^−5^	8.8015 × 10^−6^	1.5840 × 10^−5^
0.15	2.0260 × 10^−7^	6.8842 × 10^−5^	1.7456 × 10^−5^	2.9933 × 10^−5^	1.2079 × 10^−5^	1.4222 × 10^−5^
0.2	1.8344 × 10^−7^	5.8221 × 10^−5^	1.2474 × 10^−5^	3.7281 × 10^−5^	7.1484 × 10^−6^	1.3280 × 10^−5^
0.25	1.6102 × 10^−7^	6.3211 × 10^−5^	8.8643 × 10^−6^	3.7597 × 10^−5^	5.8968 × 10^−6^	1.2155 × 10^−5^
0.3	3.7975 × 10^−7^	5.1710 × 10^−5^	9.2806 × 10^−6^	3.2238 × 10^−5^	6.8494 × 10^−6^	1.1762 × 10^−5^
0.35	3.2068 × 10^−8^	7.5259 × 10^−5^	7.3542 × 10^−6^	2.4104 × 10^−5^	6.8017 × 10^−6^	1.3281 × 10^−5^
0.4	2.7219 × 10^−7^	9.0351 × 10^−5^	7.9833 × 10^−6^	1.6722 × 10^−5^	5.9056 × 10^−6^	1.2623 × 10^−5^
0.45	1.4999 × 10^−7^	7.8272 × 10^−5^	7.8305 × 10^−6^	1.4900 × 10^−5^	5.3639 × 10^−6^	1.1559 × 10^−5^
0.5	8.1631 × 10^−8^	4.4175 × 10^−5^	1.1492 × 10^−5^	1.5906 × 10^−5^	7.7188 × 10^−6^	1.1044 × 10^−5^
0.55	6.7348 × 10^−7^	4.4434 × 10^−5^	1.2222 × 10^−5^	1.8517 × 10^−5^	8.0944 × 10^−6^	1.1129 × 10^−5^
0.6	5.6913 × 10^−7^	4.5399 × 10^−5^	9.5161 × 10^−6^	2.4509 × 10^−5^	8.2024 × 10^−6^	1.1023 × 10^−5^
0.65	4.0292 × 10^−7^	5.9049 × 10^−5^	9.1083 × 10^−6^	3.1577 × 10^−5^	5.4083 × 10^−6^	9.9166 × 10^−6^
0.7	9.9417 × 10^−9^	5.7471 × 10^−5^	8.2148 × 10^−6^	3.7769 × 10^−5^	5.4840 × 10^−6^	1.1546 × 10^−5^
0.75	2.6077 × 10^−7^	5.8973 × 10^−5^	9.4506 × 10^−6^	4.0906 × 10^−5^	6.2179 × 10^−6^	1.3187 × 10^−5^
0.8	1.8827 × 10^−7^	5.3973 × 10^−5^	9.7485 × 10^−6^	3.9923 × 10^−5^	6.0827 × 10^−6^	1.1980 × 10^−5^
0.85	3.9154 × 10^−7^	4.1456 × 10^−5^	7.5585 × 10^−6^	3.5134 × 10^−5^	4.7085 × 10^−6^	8.5556 × 10^−6^
0.9	5.4210 × 10^−7^	6.6373 × 10^−5^	5.8519 × 10^−6^	2.9667 × 10^−5^	3.3879 × 10^−6^	9.4999 × 10^−6^
0.95	4.0074 × 10^−7^	6.4268 × 10^−5^	1.0509 × 10^−5^	2.7424 × 10^−5^	5.0057 × 10^−6^	1.2343 × 10^−5^
1	4.5080 × 10^−7^	3.3751 × 10^−5^	8.2858 × 10^−6^	2.8972 × 10^−5^	4.1627 × 10^−6^	8.0614 × 10^−6^

**Table 4:** Statistical presentations of the environmental and economic nonlinear model for Z(s).

s	Z(s)					
	MIN	MAX	MED	MEAN	S.I.R	STD
0	1.3315 × 10^−9^	3.1457 × 10^−5^	1.6205 × 10^−6^	3.4587 × 10^−6^	2.1651 × 10^−6^	5.1288 × 10^−6^
0.05	5.3875 × 10^−8^	2.3622 × 10^−5^	4.3838 × 10^−6^	7.8281 × 10^−6^	4.2351 × 10^−6^	5.5084 × 10^−6^
0.1	7.6305 × 10^−10^	4.2407 × 10^−5^	1.1246 × 10^−5^	1.5741 × 10^−5^	4.8690 × 10^−6^	9.0944 × 10^−6^
0.15	2.5612 × 10^−7^	7.2251 × 10^−5^	2.5533 × 10^−5^	2.9933 × 10^−5^	1.1121 × 10^−5^	1.6681 × 10^−5^
0.2	6.2359 × 10^−7^	1.0789 × 10^−4^	3.4484 × 10^−5^	3.7281 × 10^−5^	1.6162 × 10^−5^	2.2699 × 10^−5^
0.25	2.9525 × 10^−6^	1.2191 × 10^−4^	3.3155 × 10^−5^	3.7597 × 10^−5^	1.7878 × 10^−5^	2.4080 × 10^−5^
0.3	4.4801 × 10^−7^	1.1325 × 10^−4^	2.4715 × 10^−5^	3.2238 × 10^−5^	1.4273 × 10^−5^	2.2405 × 10^−5^
0.35	7.2504 × 10^−9^	8.6395 × 10^−5^	1.5573 × 10^−5^	2.4104 × 10^−5^	1.0933 × 10^−5^	1.9125 × 10^−5^
0.4	3.9256 × 10^−8^	7.9942 × 10^−5^	8.9671 × 10^−6^	1.6722 × 10^−5^	9.3565 × 10^−6^	1.6111 × 10^−5^
0.45	1.6522 × 10^−8^	6.5359 × 10^−5^	9.6346 × 10^−6^	1.4900 × 10^−5^	7.2259 × 10^−6^	1.3588 × 10^−5^
0.5	1.9922 × 10^−7^	6.3306 × 10^−5^	8.7388 × 10^−6^	1.5906 × 10^−5^	7.2795 × 10^−6^	1.2883 × 10^−5^
0.55	1.2826 × 10^−7^	8.4377 × 10^−5^	5.2377 × 10^−6^	1.8517 × 10^−5^	4.5867 × 10^−6^	1.3000 × 10^−5^
0.6	5.2687 × 10^−7^	7.7516 × 10^−5^	1.2001 × 10^−5^	2.4509 × 10^−5^	5.6850 × 10^−6^	1.0936 × 10^−5^
0.65	2.4716 × 10^−8^	5.3962 × 10^−5^	2.0504 × 10^−5^	3.1577 × 10^−5^	8.7259 × 10^−6^	1.3498 × 10^−5^
0.7	7.1897 × 10^−7^	7.8951 × 10^−5^	2.9520 × 10^−5^	3.7769 × 10^−5^	1.1816 × 10^−5^	1.9020 × 10^−5^
0.75	1.5220 × 10^−6^	1.1358 × 10^−4^	3.4899 × 10^−5^	4.0906 × 10^−5^	1.1617 × 10^−5^	2.2560 × 10^−5^
0.8	1.4351 × 10^−6^	1.4452 × 10^−4^	3.4365 × 10^−5^	3.9923 × 10^−5^	1.2464 × 10^−5^	2.4768 × 10^−5^
0.85	2.2068 × 10^−7^	1.3911 × 10^−4^	2.7204 × 10^−5^	3.5134 × 10^−5^	1.2996 × 10^−5^	2.2994 × 10^−5^
0.9	4.4985 × 10^−7^	8.8317 × 10^−5^	1.6165 × 10^−5^	2.9667 × 10^−5^	7.1411 × 10^−6^	1.6808 × 10^−5^
0.95	3.6249 × 10^−7^	6.9728 × 10^−5^	1.4044 × 10^−5^	2.7424 × 10^−5^	8.1852 × 10^−6^	1.5198 × 10^−5^
1	9.8192 × 10^−8^	5.7973 × 10^−5^	1.1354 × 10^−5^	2.8972 × 10^−5^	6.7349 × 10^−6^	1.2871 × 10^−5^

**Table 5:** Global measures performances of the environmental and economic nonlinear model.

Ω	(G-TIC)		(G-MAD)		(G-EVAF)	
	MED	S.I.R	MED	S.I.R	MED	S.I.R
X(Ω)	2.40785 × 10^−5^	1.17053 × 10^−5^	3.20176 × 10^−9^	1.48788 × 10^−9^	1.97484 × 10^−10^	1.40658 × 10^−10^
Y(Ω)	1.06193 × 10^−6^	3.97226 × 10^−6^	1.29917 × 10^−8^	4.92191 × 10^−10^	4.67588 × 10^−11^	4.10883 × 10^−11^
Z(Ω)	1.98223 × 10^−5^	6.73821 × 10^−6^	2.57316 × 10^−9^	8.41952 × 10^−10^	2.26752 × 10^−8^	1.51931 × 10^−8^

## Data Availability

Not applicable.

## Nomenclature

GAs	Genetic Algorithms
ANNs	Artificial neural networks
IPS	Interior-point scheme
FFANNs	Feed-forward ANNs
ASA	Active-set algorithm
ANN-GA-ASA	Hybrid of ANNs, GAs, and ASA
MAD	Mean absolute deviation
VAF	Variance account for
S.I.R	Semi-interquartile range
Max	Maximum
*X*	New technical diagnostics
*K_i_*	*i*th coefficient of environmental and economic indicator
*C*_1_, *C*_2_, *C*_3_	*Values of Initial conditions*
***W_X_***	*X components of W*
***W_Z_***	*Z components of W*
***w_X_***, ***w_Y_***, ***w_Z_***	*Weighting factor of X, Y, and Z*
*E*	*Fitness function*
IPA	Interior point algorithm
SQP	Sequential quadratic programming
ANN-GA-IPA	Hybrid of ANNs, GAs, and IPA
FFANN-GASQP	Hybrid of ANNs, GA, and SQP
PSO	Particle swarm optimization
ANN-PSO-IPS	Hybrid of ANNs, PSO, and IPS
TIC	Theil’s inequality coefficient
EVAF	Error in VAF
STD	Standard Deviation
Min	Minimum
*Y*	elimination costs of emergencies values
*Z*	the competence of the system of industrial elements
***W***	Known weights of ANNs or Chromosome of GAs
***W_Y_***	Y component of weights
***n_X_***, ***n_Y_***, ***n_Z_***	Scaling factors of *X*, *Y*, and *Z*
***l_X_***, ***l_Y_***, ***l_Z_***	*Biasing factor of X, Y, and Z*
*a*, *b*	*Real constant values*
